# Improvements in Wettability and Tribological Behavior of Zirconia Artificial Teeth Using Surface Micro-Textures

**DOI:** 10.3390/ma18133117

**Published:** 2025-07-01

**Authors:** Yayun Liu, Guangjie Wang, Fanshuo Jia, Xue Jiang, Ning Jiang, Chuanyang Wang, Zhouzhou Lin

**Affiliations:** 1 School of Mechanical and Electric Engineering, Soochow University, Suzhou 215021, China; yyliu6688@suda.edu.cn (Y.L.); gjwang0125@stu.suda.edu.cn (G.W.); 20235229047@stu.suda.edu.cn (F.J.); 20235229084@stu.suda.edu.cn (X.J.); jiangning@suda.edu.cn (N.J.); 2 School of Politics and Public Administration, Soochow University, Suzhou 215123, China

**Keywords:** tribological behavior, surface micro-textures, surface wettability, artificial teeth

## Abstract

Zirconia ceramics are promising materials for restoration and are widely used in the field of artificial teeth. However, wear resistance affects the longevity of artificial teeth. In this study, peacock tail feather micro-textures and groove micro-textures are prepared on the surfaces of zirconia ceramics via the laser ablation technique to improve their tribological properties. The effects of micro-textures on the surface wettability and tribological properties of zirconia ceramics are studied. The micro-textures improve the surface wettability and tribological properties of zirconia ceramics. The average coefficient of friction of peacock tail feather micro-textured samples decreases by 53% compared to that of the samples without micro-textures. Different operating conditions affect the friction properties of zirconia ceramics. The samples have the best friction performance when the rotational speed, load, and acid/alkaline environment are 200 r/min, 15 N, and weakly alkaline, respectively. Furthermore, the mechanism by which surface micro-textures reduce frictional wear is as follows: the textured grooves store debris, and the bottom edge of the textured groove acts as a cutting tool to cut debris, preventing debris from scratching the surface. The micro-textures store lubricant and form a liquid film on the ceramic surface to reduce wear.

## 1. Introduction

Teeth play a critical role in both esthetics and masticatory function [1,2]. To address the growing demand for durable and esthetically pleasing dental solutions, restorative dentistry has become a cornerstone of modern dental care. Different types of materials are studied as candidates for dental restorations. Mechanical integrity, corrosion, biocompatibility, friction, wear, and esthetics are some of the key considerations [3]. Metallic materials have excellent mechanical and corrosion resistance properties. However, they are less biocompatible and prone to micro-scale wear. Composites are introduced as dental implant materials because of their good biocompatibility and esthetic properties. However, their excessive wear is the greatest challenge in the use of these materials. Zirconia ceramics are gradually replacing traditional composites and metals in the manufacturing of dental implants due to their good mechanical properties, excellent wear resistance, and biocompatibility [4]. Among them, yttria-stabilized zirconia (YSZ) demonstrates particularly outstanding performance. Researchers investigated the properties of YSZ nanocomposites doped with 3 mol% alumina under simulated artificial saliva conditions. The results showed that YSZ exhibited excellent chemical stability and mechanical property durability by maintaining crystal phase stability and inhibiting low-temperature degradation in an artificial saliva environment [5]. Another group of researchers focused on the electrochemical behavior of dental zirconia ceramics in acidic environments. It was shown that YSZ exhibited a low corrosion rate, an oxide film protection mechanism, and crystalline phase stability under acidic conditions. This suggests the potential for the long-term chemical stability of YSZ in an artificial saliva environment [6]. The optical behavior of the YSZ samples is a crucial property, especially in the context of dental applications, where esthetics is important. Researchers investigated the optical properties of yttria-stabilized tetragonal zirconia polycrystals (Y-TZP) for dental ceramics. The results demonstrated that increasing the light transmittance to over 70% led to optical properties that closely approximated the optical performance of natural teeth. A simultaneous improvement in optical stability and long-term clinical applicability can be achieved through compositional gradient design and crystal boundary modulation [7,8].

Dental materials are inevitably subjected to reciprocal friction from the teeth. Therefore, the wear and tear of artificial teeth is an inevitable process [9,10]. Excessive wear of dental restorations can disrupt the occlusal harmony, resulting in diminished masticatory efficiency and compromised esthetic outcomes [11]. Therefore, it is necessary to evaluate the tribological properties of dental restorative materials in clinical applications. P Pinto et al. evaluated the wear resistance of four dental restorative materials against toothbrush abrasion [12]. Zirconia was found to have the best wear resistance: about 10 times that of feldspathic ceramics and composites and about 100 times that of polymers. Amanda Carvalho et al. compared the wear properties of major dental restorative materials (polymers, composites, and ceramics), with zirconia ceramics demonstrating the highest wear resistance [13]. M. S. Zafar et al. compared the wear behavior of various dental restorative materials and the effects of wear on the surface mechanical properties [14]. Dental ceramics showed the most remarkable wear resistance. Recently, zirconia ceramics have become the most commonly used dental restorative material. Despite their wide application, the high hardness of zirconia ceramics brings new challenges. Due to the inherently high hardness of zirconia ceramics, they are prone to excessive wear on natural teeth during occlusion. Excessive wear has become a major problem for ceramic restorations in clinical applications [15].

Surface micro-textures are an effective way to reduce friction and wear at contact interfaces [16,17]. Numerous experimental studies have been carried out to investigate the laser treatment of zirconia ceramic surfaces. J. Wang et al. analyzed the effects of surface micro-textures on the friction properties of Cr12MoV [18]. Surface micro-textures were found to be effective in improving the surface roughness and reducing the coefficient of friction. J. Sun et al. investigated the effects of surface micro-textures on the friction behavior of bearing replacements [19]. The samples with micro-textures were found to have the best friction reduction and wear resistance, followed by smooth samples, and the worst were rough samples. L. Lu et al. investigated the effects of laser surface micro-textures on friction and wear behavior [20]. Laser surface micro-textures were found to significantly reduce the wear volume and improve the surface properties of chrome alloys. The effects of micro-textures on surface friction and wear properties were analyzed by Z. Wu et al. Micro-textures were found to be effective in reducing the coefficient of friction and its fluctuations [21]. These studies on various materials have demonstrated the potential of surface micro-textures in enhancing the tribological properties, but their application in dental restorative materials remains underexplored. In the current friction research, surface micro-textures have been shown to reduce the surface friction coefficients. However, there is very little research into improving the tribological properties of dental restorative materials using surface micro-textures.

Therefore, in this study, zirconia ceramics are chosen as the substrate for artificial teeth. To improve the tribological properties of artificial teeth, two different types of micro-textures (groove micro-textures and peacock tail feather micro-textures) are processed on the surfaces of zirconia ceramics by laser machining. We explore the influence of micro-textures on the surface wettability and friction properties of zirconia artificial teeth. We investigate the effects of the load, acid–base environment, and rotational speed on the tribological properties of artificial teeth. This paper describes the mechanisms by which micro-textures affect the surface wettability and tribological properties of zirconia ceramics, aiming to provide theoretical guidance for the optimization of the performance of artificial teeth.

## 2. Experimental Methods

### 2.1. Zirconia Artificial Teeth Ceramic Specimens and Surface Texture Processing

In this study, 3Y-TZP is selected as the substrate for artificial teeth. Zirconia ceramics have exceptional mechanical properties, excellent wear resistance, and biocompatibility. The composition and physical properties of the zirconia ceramics are shown in Table 1 and Table 2. To meet the clamping requirements of the friction test rig, samples are cut into square blocks with a side length of 25 mm and a thickness of 3 mm. Two types of surface micro-textures are designed in a 20 mm × 20 mm area on the surfaces of the samples. The studied texture patterns include groove micro-textures and peacock tail feather micro-textures. The prepared zirconia ceramics are cleaned with alcohol for 30 min prior to surface texturing.

**Figure 1:**
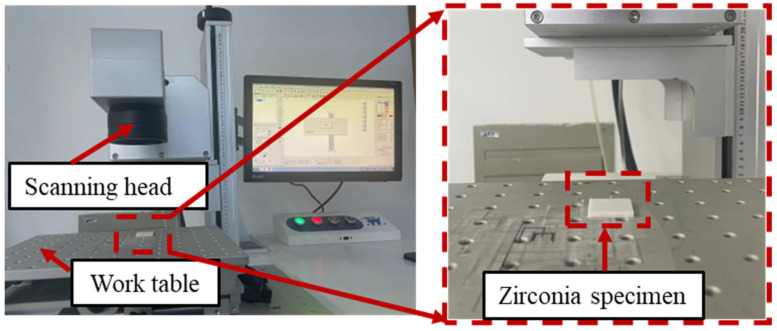
The experimental setup of the laser surface texturing process.

The zirconia ceramic surface micro-textures are processed by laser (ZT-F05). The experimental setup is shown in Figure 1. The picosecond laser has maximum power of 15 W, a wavelength of 355 nm, and a rated frequency of 50 Hz. The processing parameters for laser processing are as follows: laser frequency *f* = 30 kHz, laser power P = 5 W, scanning speed v = 60 mm/s, and the number of scans is 10.

**Figure 2:**
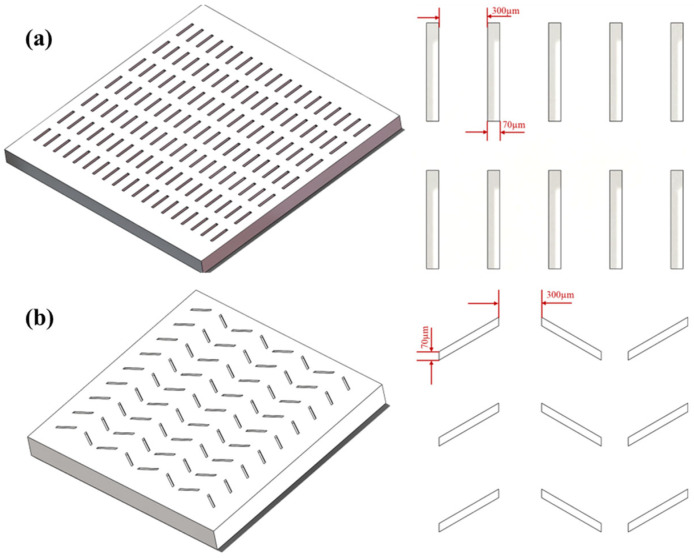
Schematic and detailed geometric characterization of zirconia ceramic surface micro-textures. (**a**) MT-1, (**b**) MT-2.

**Figure 3:**
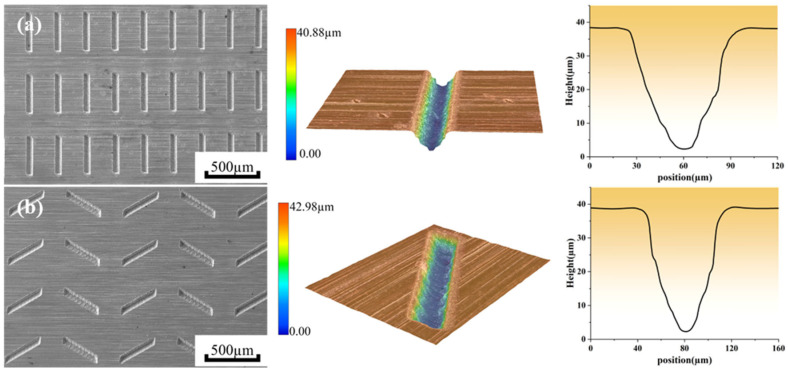
Surface morphologies and profiles of zirconia ceramics with different micro-textures. (**a**) MT-1, (**b**) MT-2.

The groove micro-textures are named MT-1 and the peacock tail feather micro-textures are named MT-2. Zirconia ceramics without micro-textures are named MT-0. Figure 2 shows a schematic diagram and the detailed geometric features of the zirconia ceramic surface micro-textures. Figure 3 shows the surface morphologies and contours of zirconia ceramics with different micro-textures. The dimensional parameters of both the groove micro-textures and the peacock tail feather micro-textures are as follows: 300 μm spacing, 70 μm width, 45 μm depth. Some previous studies in the literature suggest that linear grooves with a pitch of 100–300 μm, a depth of 30–100 μm, and a width of about 20–50 μm show reduced friction and improved wear resistance [22]. Here, the zirconia ceramics are cleaned with alcohol for 15 min prior to friction and wear testing.

### 2.2. Wettability Test

**Figure 4:**
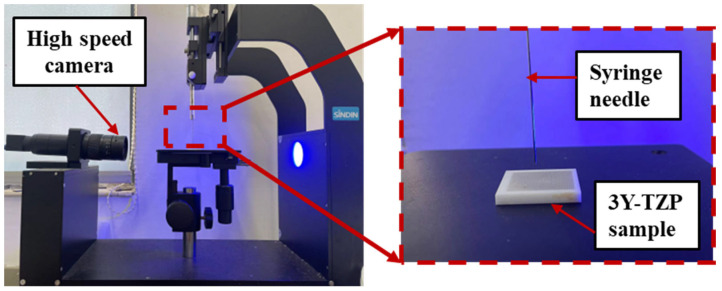
The measurement of the contact angle for a micro-textured zirconia specimen.

Wettability is an important property characterizing the interaction between solid surfaces and liquids. The surface wettability is measured by a contact angle meter (SDC-100). A diagram and schematic of the device used to measure the surface wettability are shown in Figure 4. During the test, 10 µL of deionized water is dropped onto the zirconia ceramic surface. The contact angle is calculated automatically using an optical contact angle measuring instrument. To ensure reliability, each contact angle measurement is repeated three times and the average value is calculated.

### 2.3. Friction Test

**Figure 5:**
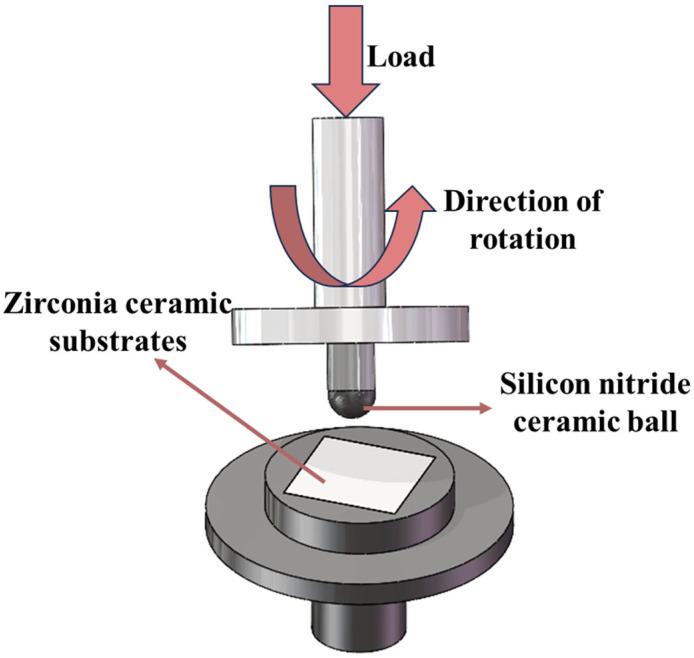
Schematic diagram of zirconia artificial tooth friction and wear experiment.

Friction tests are conducted to evaluate the tribological behavior of the micro-textured zirconia ceramics. The friction and wear performance testing equipment in this experiment adopts an MMW-1D multi-function universal friction and wear tester. The experimental setup is shown in Figure 5. In the friction experiments, the ball specimens are silicon nitride ceramic balls with a diameter of 6 mm, and the disc specimens are zirconia ceramic substrates with a side length of 25 mm and a thickness of 3 mm. Tests are performed on zirconia ceramics in artificial saliva. Steps for creation of artificial saliva: firstly, add an appropriate amount of deionized water into the container; then, slowly add magnesium hexahydrate, calcium chloride dihydrate, sodium chloride, potassium chloride, dipotassium hydrogen phosphate trihydrate, potassium carbonate, and sodium carboxymethylcellulose and stir to dissolve them completely. Afterwards, adjust the pH of the above solutions to 6, 7, and 8 with a hydrochloric acid solution. After the friction test, the wear patterns on the surfaces of the micro-textured zirconia ceramics are characterized by SEM (VEGA3 XMH).

## 3. Results and Discussion

### 3.1. Wettability Analysis

**Figure 6:**
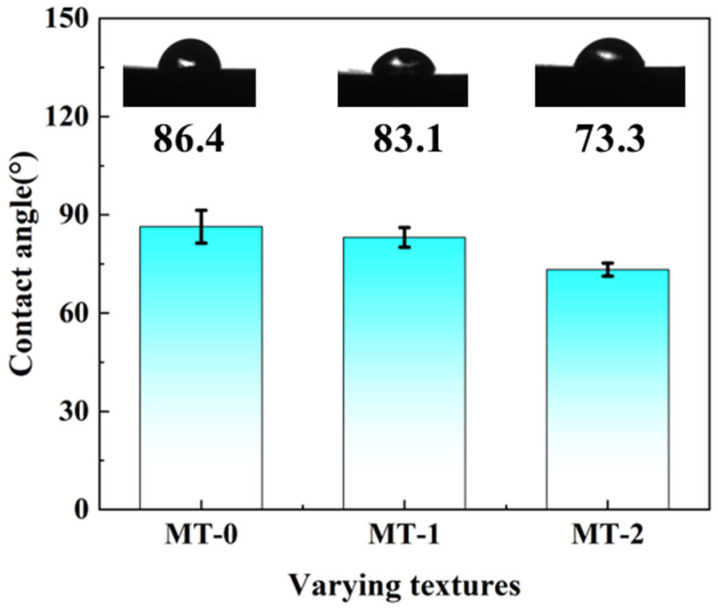
Contact angles of different micro-textured zirconia surfaces.

Surface wettability is an important property characterizing the interaction between solid surfaces and liquids [23]. Surface micro-textures can alter the wettability of a material, thus affecting its hydrophobic or hydrophilic properties. The contact angle is an important indicator in assessing surface wettability. Therefore, the contact angle is used to evaluate the surface wettability of zirconia ceramics, and the results are shown in Figure 6.

Figure 6 shows the variation in the contact angle with different surface micro-textures. It can be concluded that micro-textures improve the surface wettability of zirconia ceramics. As illustrated in Figure 6, the contact angle of MT-0 (86.4°) indicates hydrophilic behavior, while MT-1 and MT-2 exhibit lower values (83.1° and 73.3°), confirming the enhanced wettability via micro-textures.

From the above analysis, it is concluded that micro-textures improve the surface wettability of zirconia ceramics. MT-2 has the smallest contact angle and therefore the best hydrophilicity.

### 3.2. Friction and Wear Performance Analysis

#### 3.2.1. Effects of Texture on Frictional Properties of Zirconia Artificial Teeth

**Figure 7:**
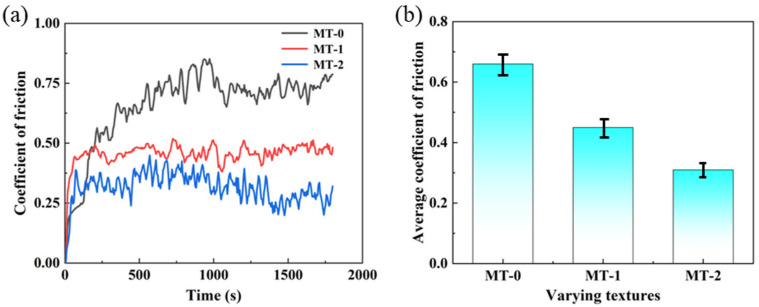
Frictional properties of different textured zirconia artificial teeth surfaces. (**a**) Friction coefficient, (**b**) average friction coefficient.

Friction experiments are conducted on the zirconia ceramic surfaces under artificial saliva lubrication conditions. Figure 7a shows the coefficient of friction curves for different micro-textured surfaces under artificial saliva lubrication conditions. Figure 7b shows the average friction coefficients for different micro-textured surfaces.

As shown in Figure 7a, the friction coefficient curve of MT-0 varies greatly. The friction curve shows an increasing trend in the first 15 min and then remains stable. MT-1 and MT-2 have a lower coefficient of friction and show less fluctuation compared to MT-0. MT-1 has a relatively higher coefficient of friction compared to MT-2 but shows significantly smaller curve fluctuations. MT-2 has the lowest coefficient of friction and less overall fluctuation. As shown in Figure 7b, MT-0 has the largest average coefficient of friction at 0.66. MT-2 has the smallest average friction coefficient of 0.34. The average friction coefficient of MT-2 is 53% lower than that of MT-0. This is followed by MT-1, which reduces the average coefficient of friction by 31%. It can be concluded that the surface micro-textures significantly reduce the coefficient of friction. This is because the micro-textures reduce the contact area of the friction pairs and store wear debris, and the debris further protects the textured grooves from wear and tear [24,25].

**Figure 8:**
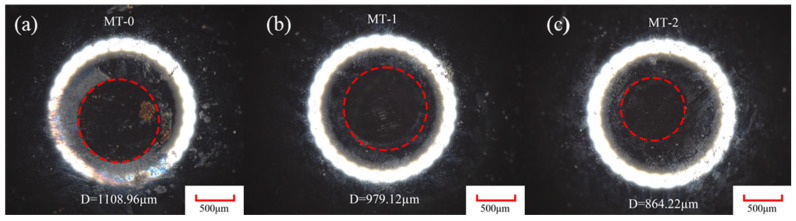
Wear diameters of different micro-textured silicon nitride ceramic balls, D. (**a**) MT-0, (**b**) MT-1, (**c**) MT-2.

In vitro friction experiments simulate the friction and wear relationship between occlusal teeth during masticatory movements. Therefore, the amount of wear on the samples also indicates tribological relationships. Figure 8 shows the wear surfaces of silicon nitride ceramic balls with different micro-textures, as well as the wear diameter D. As shown in Figure 8, the surface micro-textures are effective in reducing the amount of wear on the ceramic balls. MT-0 shows the greatest wear of the silicon nitride ceramic balls, with a wear diameter of 1108.96 μm. MT-2 exerts the least wear on the silicon nitride ceramic balls, with a wear diameter of 864.22 μm. Compared to MT-0, the wear diameter D for the MT-1 and MT-2 ceramic balls is reduced by 11.7% and 22%, respectively.

**Figure 9:**
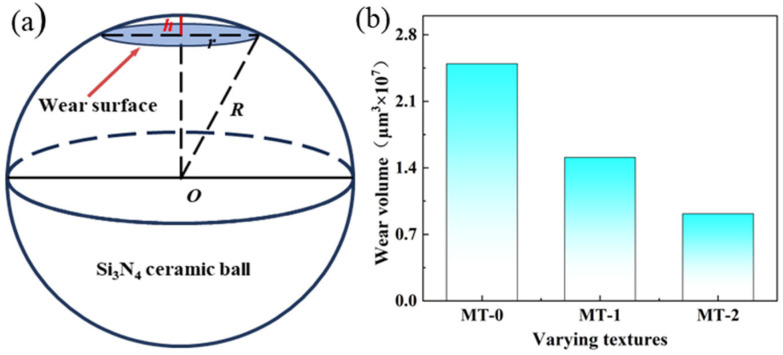
Effects of different textures on wear volume. (**a**) Wear model, (**b**) wear volume.

To further quantify the effectiveness of the surface micro-textures, the wear resistance of different micro-textures is assessed by calculating the wear volume loss. The wear volume loss is calculated using the wear spherical crown, shown in Figure 9a.

The amount of ball wear can be expressed as

(1) $$V=\frac{\pi h^{2}(3r-h)}{3}$$

where *r* is the radius of the spherical crown and h is the height of wear.

(2) $$h=R-\sqrt{R^{2}-r^{2}}$$

where *R* is the radius of the ceramic ball.

Based on the calculated wear volume loss, a comparison of the wear volumes among different samples is presented as follows. MT-0 has the largest wear volume of 2.5 × 10^7^ μm^3^ under artificial saliva lubrication conditions. MT-2 has the smallest wear volume of 9.18 × 10^6^ μm^3^. This is followed by the wear volume of MT-1, which is 1.51 × 10^7^ μm^3^. Compared to MT-0, the wear volumes of the MT-1 and MT-2 ceramic balls are reduced by 39.6% and 63.2%, respectively. This shows that the micro-textures are effective in reducing the wear of silicon nitride ceramic balls, and this is because the micro-textures’ grooves can store the debris generated by friction, preventing it from scattering in the friction area, thus further protecting the textured grooves from wear and tear.

#### 3.2.2. Effects of Different Parameters on Friction Characteristics of Zirconia Artificial Teeth

**Figure 10:**
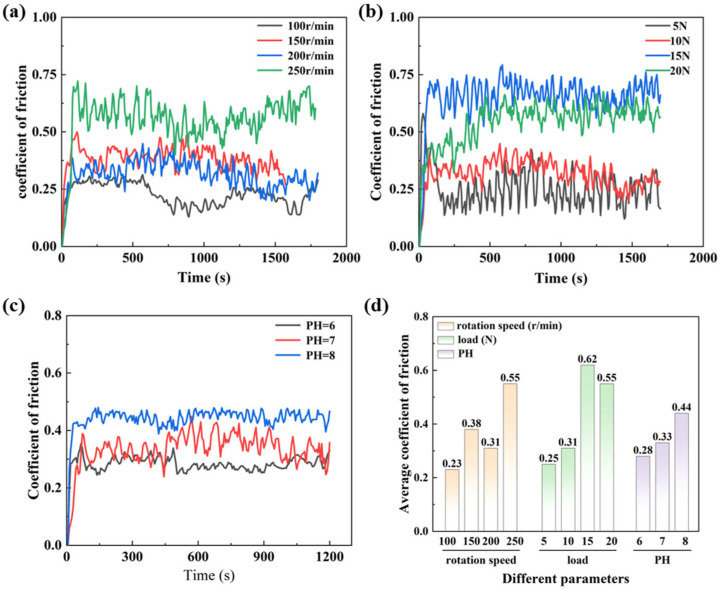
Effects of different parameters on the coefficients of friction of zirconia ceramics. (**a**) Rotation speed, (**b**) load, (**c**) pH, (**d**) average friction coefficient for different parameters.

Figure 10a shows the coefficient of friction curves of MT-2 at 100 r/min, 150 r/min, 200 r/min, and 250 r/min. The friction coefficient reaches a minimum at 100 r/min, with generally minor fluctuations, whereas it peaks at 250 r/min, accompanied by the most significant curve variations. At 200 r/min, the coefficient of friction is smaller and the fluctuation of the curve is smaller. As shown in Figure 10d, as the speed increases from 100 r/min to 200 r/min, the average coefficient of friction increases and then decreases with increasing speeds. The average coefficient of friction increases when the speed is gradually increased from 200 r/min to 250 r/min.

Load variations lead to different wear mechanisms in zirconia materials, affecting their life. Figure 10b shows the coefficient of friction of MT-2 at loads of 5 N, 10 N, 15 N, and 20 N. At a load of 5 N, the coefficient of friction is minimized but the curve fluctuates. The highest coefficient of friction occurs at a load of 15 N. At a load of 10 N, the coefficient of friction is low and the curve fluctuates little.

As shown in Figure 10d, the average coefficient of friction increases and then decreases with increasing loads from 5 N to 20 N. The average coefficient of friction is the maximum at 15 N. This is due to the fact that the increased load may render the grooves on the surface more likely to be filled with lubricant [26]. This facilitates the formation of a lubricating film and reduces friction.

Figure 10c shows the friction coefficient curves and the variation in the average friction coefficient of MT-2 under different acidic and alkaline environments. It can be seen that acidity and alkalinity have a significant effect on the frictional properties of the prepared artificial teeth. As shown in Figure 10c, the coefficient of friction is the minimum and the curve fluctuates the least when the environment is weakly acidic, but the curve has a significant drop at 500 s. The coefficient of friction is the greatest and the curve fluctuates the most steadily when the environment is weakly alkaline. When the environment is neutral, the friction coefficient is smaller but the curve fluctuates more sharply. As shown in Figure 10d, the average coefficient of friction tends to increase slowly with the increasing pH in the experiment. This phenomenon is due to the fact that H⁺ in the weakly acidic solution slightly dissolves the Y_2_O_3_ stabilizer on the surface of the zirconia ceramic, inducing a phase change in the surface layer. It effectively stores lubricant and reduces the direct contact area of the friction partners. In weakly alkaline environments, the stability of the original lubrication film on the ceramic surface may be compromised, thereby diminishing the interfacial lubrication effect.

#### 3.2.3. Surface Morphology Analysis of Zirconia Artificial Teeth

**Figure 11:**
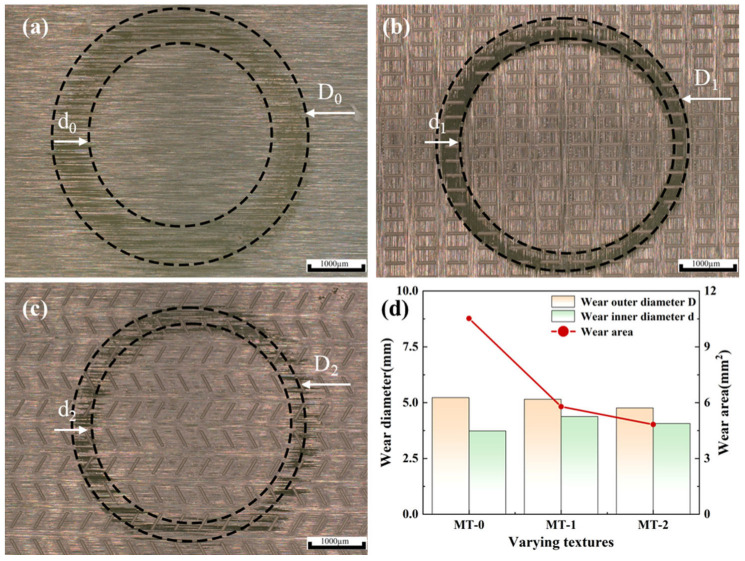
Wear regions and wear areas of zirconia ceramics with different micro-textures. (**a**) MT-0, (**b**) MT-1, (**c**) MT-2, (**d**) wear diameter and wear area with different textures.

Figure 11 shows the wear characteristics of zirconia ceramics with different micro-textures. As shown in Figure 11d, MT-0 has the largest surface wear area. This is due to friction between the silicon nitride ceramic balls and the zirconia ceramic substrate. The generated wear debris is distributed on the surface of the wear area; it then enters the friction area and participates in friction. This leads to increased wear. The surfaces of MT-1 and MT-2 have a narrower wear width and lower wear volume compared to MT-0. MT-2 has the lowest wear level.

**Figure 12:**
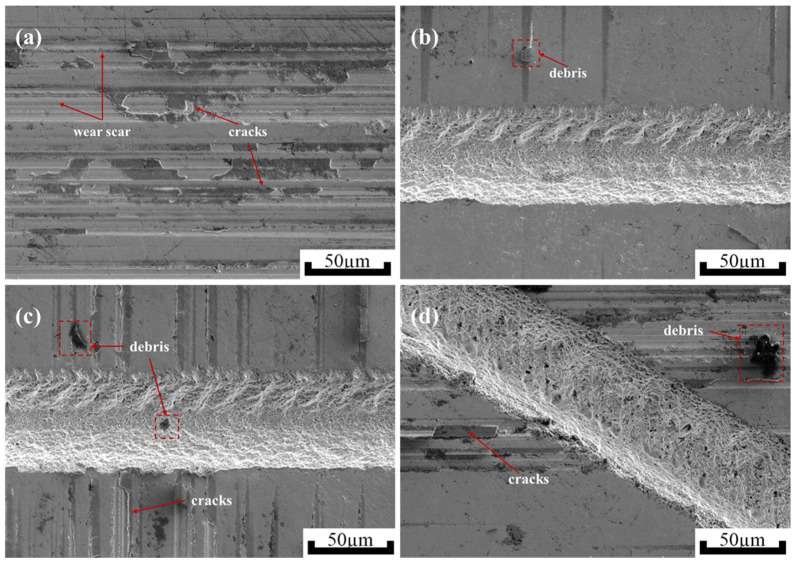
Micrographs of different micro-textured zirconia ceramic surfaces before and after friction. (**a**) Without texture after friction, (**b**) microgroove texture before friction, (**c**) microgroove texture after friction, (**d**) peacock tail feather texture after friction.

To further determine the effects of micro-textures on the friction and wear behavior of zirconia ceramic samples, SEM is carried out on the wear traces on the surfaces of the zirconia ceramics. Figure 12 shows micrographs of different micro-textured zirconia ceramic surfaces before and after friction. Combining Figure 12b,c, it is concluded that the overall ceramic surface is relatively flat before friction. Only a small amount of residue is present on the surface. This is probably due to the laser texturing process. After friction, the ceramic surface shows obvious wear marks. Cracks on the surface of the ceramic and the presence of debris flaking off from the silicon nitride spheres are observed. Combining the wear areas in Figure 11 and the microscopic morphology of Figure 12, it is found that the wear on the surfaces of zirconia ceramics without micro-textures are greater than in those with micro-textures. A large amount of wear debris can be seen adhering to the wear surface of the non-textured zirconia ceramic. These fragments can damage the ceramic surface in subsequent friction and produce cracks. It can be seen that micro-textured ceramics produce debris that is distributed not only on the surface but also within the textured grooves. Wear is reduced by storing the debris. On the one hand, this prevents the debris from being involved in subsequent friction to damage the ceramic surface. On the other hand, debris accumulated at the bottom edges of textured grooves serves as an abrasive, shearing the wear particles adhering to the silicon nitride balls. Overall, these observations indicate that the surface micro-textures effectively reduce the friction in the zirconia ceramic specimens.

### 3.3. Mechanistic Analysis of Micro-Textures and Friction Performance

In this paper, the effects of micro-textures on the wear behavior of zirconia artificial teeth have been investigated. The results show that surface micro-textures significantly improve the wear resistance of the prepared zirconia artificial teeth. To further determine the effects of micro-textures on the friction and wear behavior of zirconia artificial teeth specimens, the responsible mechanisms are discussed below.

**Figure 13:**
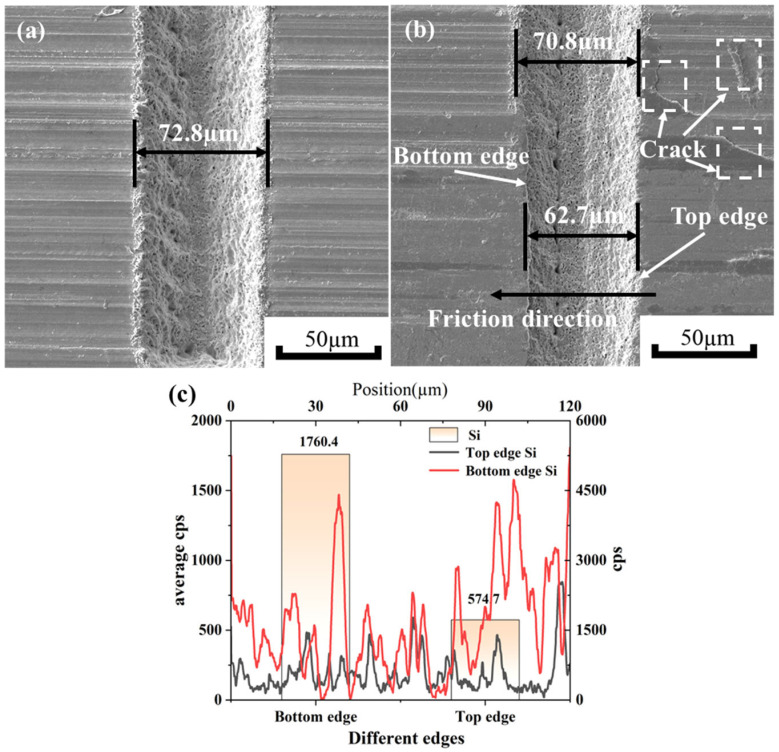
Microscopic morphologies of micro-textured grooves before and after friction and analysis of Si elements at different positions. (**a**) Microscopic morphology of textured grooves before friction, (**b**) microscopic morphology of textured grooves after friction, (**c**) silicon content at different edges of the element.

**Figure 14:**
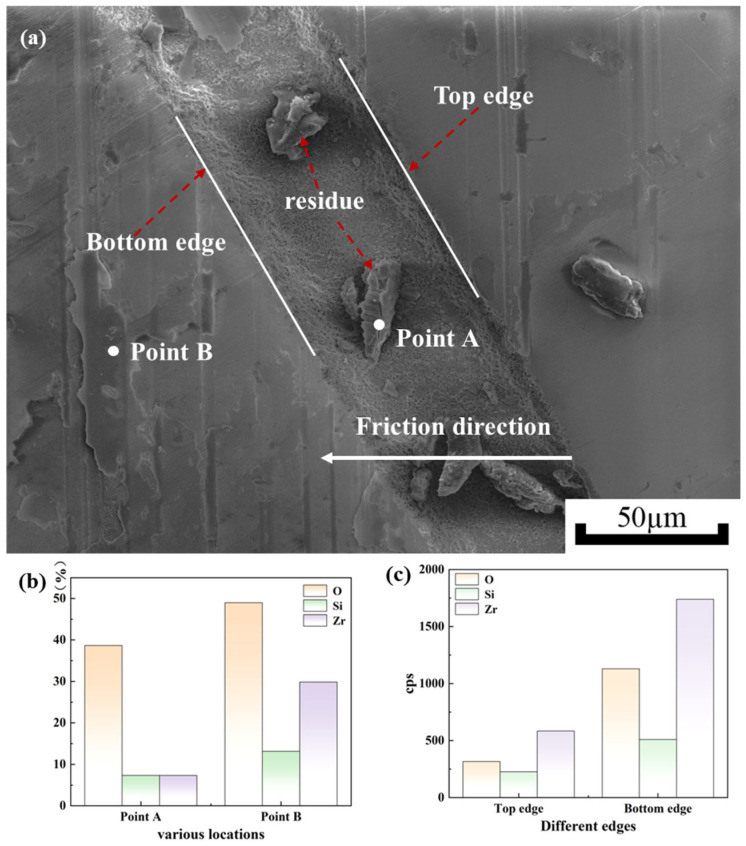
Surface morphologies of textured grooves and analysis of elements at different locations. (**a**) Surface morphologies of textured grooves, (**b**,**c**) elemental analyses in different locations.

SEM micrographs of the micro-texture grooves are shown in Figure 13. As illustrated in Figure 13a, no ablation-induced cracks are observed around the grooves after laser processing. In contrast, Figure 13b reveals friction-generated cracks around the grooves post-friction. As can be seen in Figure 13b, the width of the textured groove after laser processing is 72.8 µm. After friction, the width of the textured grooves is 70.8 µm in one section and 62.7 µm in another section. It can be concluded that the bottom edge of the texture is heavily abraded, causing the Si element from the silicon nitride ceramic spheres to adhere to it. Figure 14 shows the SEM micrographs of the micro-texture grooves and the results of their EDX surface chemistry analysis. The EDS analysis of the residues reveals that they contain a significant amount of Si. This indicates that there is debris from the silicon nitride ceramic balls remaining in the textured grooves. It is proven that derivative cutting occurs at the bottom edge of the texture. Derivative cutting is the additional cutting of debris between the silicon nitride ceramic balls and the micro-textures on the surface of the zirconia ceramic substrate. The bottom edge of the texture serves as a ‘cutting tool’ to remove debris adhering to the silicon nitride ceramic balls during friction [27,28]. This cutting avoids damage to the zirconium oxide surface by debris. By comparing the micrographs of the micro-textures’ grooves shown in Figure 13, it can be concluded that the aggregation of Si elements at the bottom edge of the texture, as well as the change in the width of the textured grooves, is caused by the phenomenon of derivative cutting.

As shown in Figure 14a, it can be seen that the wear around the bottom edge of the textured groove is greater than around the top edge. There are many residues in the texture grooves. EDS analysis is performed on the residues in the textured grooves and the debris around the bottom edge to clarify their elemental compositions. As shown in Figure 14b, an elemental comparison of points A and B reveals that point B around the bottom edge has higher content of both Si and Zr elements than point A. Combined with the surface morphology, these results indicate more severe wear at point B, leading to the exposure of the zirconia ceramic substrate. Therefore, high content of Zr elements is detected at point B, which corresponds to the results shown in Figure 14b. EDS analysis is conducted on the top and bottom edges of the textured grooves to determine their elemental compositions. As illustrated in Figure 14c, the content of both Si and Zr elements at the bottom edge is found to be higher than that at the top edge. This corresponds to the conclusion drawn from the observations in Figure 13, demonstrating that derivative cutting occurs at the bottom edge.

Micro-textures on zirconia ceramics improve their surface wettability. Surface wettability affects the flow of the lubricant and helps to reduce surface wear and the coefficient of friction. To reveal the mechanism of the micro-texture’s influence on the surface wettability, the classical wettability model, the Wenzel model, is introduced [29].

(3) $$\cos\theta_{i}=\eta \cos\theta_{\lambda }$$

where $\theta_{\lambda }$ denotes the contact angle for smooth surfaces and $\theta_{i}$ denotes the contact angle for microtextured surfaces. η indicates the value of the rough surface area divided by the projected surface area.

**Figure 15:**
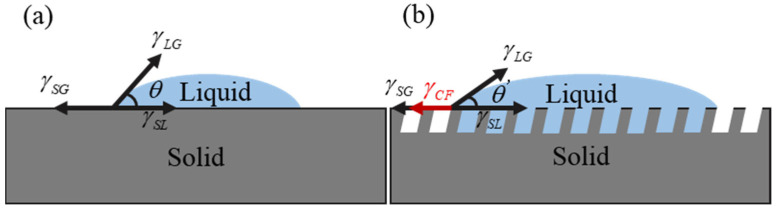
Schematic diagram of different surface wettability. (**a**) Without micro-textured surface, (**b**) micro-textured surface.

Micro-textured grooves have a capillary attraction to liquids on the surface [30]. Thus, when evaluating the surface wettability of micro-textured zirconia ceramics, the capillary action of the grooves must be taken into account. Capillary attraction keeps the droplet in a stress equilibrium, as shown in Figure 15. Therefore, the contact angle of the micro-textured surface can be obtained by the following equation:

(4) $$\gamma_{SG}+\gamma_{CF}=\gamma_{SL}+\gamma_{LG}\cos\theta \prime$$

$\gamma_{CF}$ is the capillary attraction of the micro-texture grooves to the liquid. $\gamma_{SG}$, $\gamma_{SL}$, and $\gamma_{LG}$ represent the interfacial tension of gas–solid, solid–liquid, and gas–liquid, respectively.

Based on the foregoing analysis, it can be concluded that the contact angle $\theta^{\prime }$ of the lubricant on the micro-textured surface is smaller than that of the smooth surface θ. The smaller the contact angle of a liquid, the more hydrophilic it is and the more it can diffuse [31]. As a result, the lubricant spreads more easily over the micro-textured surfaces. This suggests the better surface wettability of zirconia ceramics with micro-textured surfaces. Enhanced surface wettability improves the friction performance. This improvement is attributed to the fact that the excellent wettability of micro-textures facilitates lubricant film formation on the friction contact surface, effectively reducing the friction coefficient.

**Figure 16:**
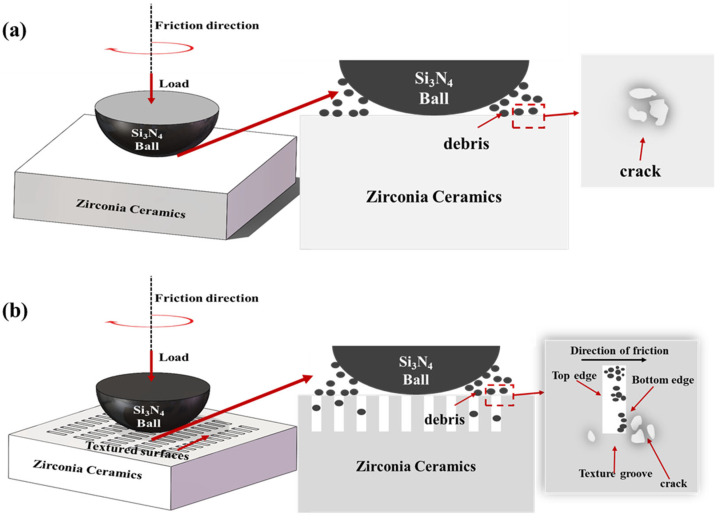
Schematic representation of the effects of surface textures on the friction behavior between zirconia artificial teeth. (**a**) Without micro-textures, (**b**) micro-textures.

Based on the analytical and experimental results, Figure 16 depicts the effects of micro-textures on the friction behavior of zirconia artificial teeth. This paper explains the mechanisms responsible for the enhanced frictional properties of micro-textured zirconia artificial teeth as follows: firstly, the micro-textures reduce wear by storing wear debris and preventing it from engaging in further friction; secondly, derivative cutting occurs at the bottom edge of the textured groove. The bottom edge cuts debris attached to the silicon nitride spheres, thereby reducing the wear on the zirconia ceramic surfaces.

## 4. Conclusions

In this paper, the effects of micro-textures on the tribological behavior of zirconia artificial teeth is investigated via in vitro friction experiments. Multi-perspective analyses of the contact angle, friction coefficient, wear volume, and wear surface morphology indicate that micro-texturing is beneficial for the frictional properties of artificial teeth. The specific conclusions of this paper are as follows:

(1) Zirconia artificial teeth with micro-textures exhibit better friction properties compared to MT-0. MT-2 has the best friction properties. The average coefficient of friction is the smallest at 0.34.
(2) Micro-textures improve the frictional properties of zirconia artificial teeth under different working conditions. The coefficient of friction increases as the pH of the lubricant increases. Moreover, as the load increases, the coefficient of friction first increases and then decreases, reaching its peak at 15 N. Additionally, zirconia artificial teeth demonstrate the optimal friction performance at a rotational speed of 200 r/min.
(3) Micro-textures reduce the coefficient of friction by storing debris and lubricant during friction. The bottom edge of the textured grooves acts as a cutting tool to cut debris adhering to the silicon nitride ceramic balls, preventing debris from scratching the surface. The surface contact angles of MT-1 and MT-2 are 83.1° and 73.3°, respectively, which are smaller than that of MT-0. The surfaces of zirconia ceramics are more hydrophilic due to the micro-textures. The hydrophilic surface forms a lubricating film on the ceramic surface, thus reducing the coefficient of friction.

## Figures and Tables

**Table 1:** Composition of zirconia ceramics.

Element	ZrO_2_	Y_2_O_3_	Al_2_O_3_	SiO_2_	TiO_2_
Quantity contained (%)	≥94	5.33	0.23	0.007	0.0012

**Table 2:** Physical properties of the used zirconia ceramics.

Physical Property	Value	Physical Property	Value
Density/(kg/m^3^)	6000	Thermal conductivity/(W/(m·k))	2.5
Poisson’s ratio	0.23	Durometer/HV	1200
Bending strength/MPa	1000	Compressive strength/MPa	2100
Young’s modulus/GPa	210	Rupture strength/MPa	800

## Data Availability

The original contributions presented in this study are included in the article. Further inquiries can be directed to the corresponding authors.
